# Desirable Traits of a Good Biocontrol Agent against Verticillium Wilt

**DOI:** 10.3389/fmicb.2017.01186

**Published:** 2017-07-06

**Authors:** Silke Deketelaere, Lien Tyvaert, Soraya C. França, Monica Höfte

**Affiliations:** Laboratory of Phytopathology, Department of Crop Protection, Faculty of Bioscience Engineering, Ghent UniversityGhent, Belgium

**Keywords:** biocontrol, biological control, cross-protection, endophytes, soil-borne pathogens, survival structures, vascular pathogen, Verticillium wilt

## Abstract

The soil-borne fungus *Verticillium* causes serious vascular disease in a wide variety of annual crops and woody perennials. Verticillium wilt is notoriously difficult to control by conventional methods, so there is great potential for biocontrol to manage this disease. In this study we aimed to review the research about *Verticillium* biocontrol to get a better understanding of characteristics that are desirable in a biocontrol agent (BCA) against Verticillium wilt. We only considered studies in which the BCAs were tested on plants. Most biocontrol studies were focused on plants of the *Solanaceae, Malvaceae*, and *Brassicaceae* and within these families eggplant, cotton, and oilseed rape were the most studied crops. The list of bacterial BCAs with potential against *Verticillium* was dominated by endophytic *Bacillus* and *Pseudomonas* isolates, while non-pathogenic xylem-colonizing *Verticillium* and *Fusarium* isolates topped the fungal list. Predominant modes of action involved in biocontrol were inhibition of primary inoculum germination, plant growth promotion, competition and induced resistance. Many BCAs showed *in vitro* antibiosis and mycoparasitism but these traits were not correlated with activity *in vivo* and there is no evidence that they play a role *in planta*. Good BCAs were obtained from soils suppressive to Verticillium wilt, disease suppressive composts, and healthy plants in infested fields. Desirable characteristics in a BCA against *Verticillium* are the ability to (1) affect the survival or germination of microsclerotia, (2) colonize the xylem and/or cortex and compete with the pathogen for nutrients and/or space, (3) induce resistance responses in the plant and/or (4) promote plant growth. Potential BCAs should be screened in conditions that resemble the field situation to increase the chance of successful use in practice. Furthermore, issues such as large scale production, formulation, preservation conditions, shelf life, and application methods should be considered early in the process of selecting BCAs against *Verticillium*.

## Introduction

**Figure 1:**
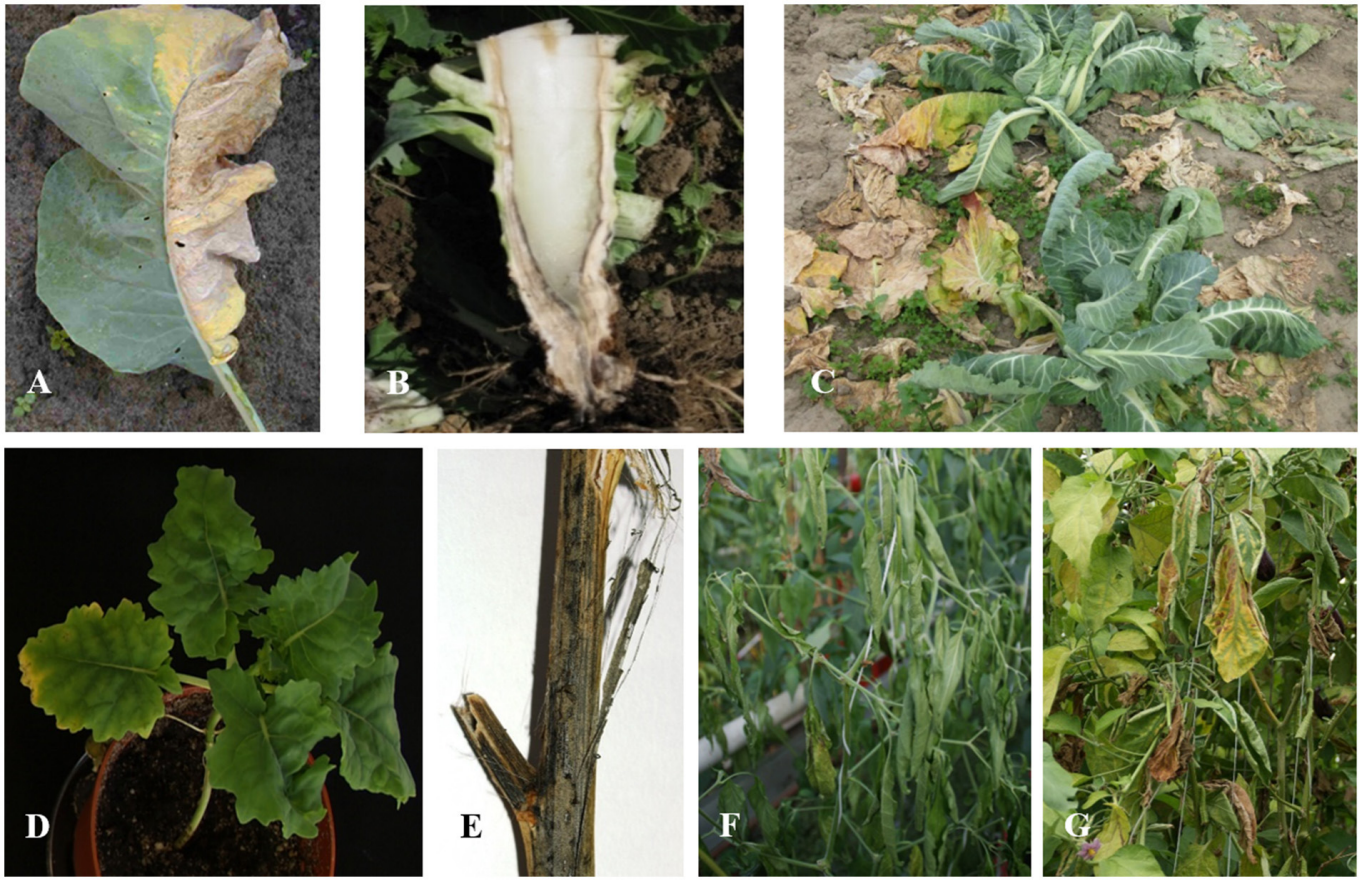
Symptoms caused by *Verticillium* spp. Verticillium wilt of cauliflower **(A–C):** Asymmetric chlorosis of the leaves **(A)**; Vascular discoloration of the stem **(B)**; Wilting of cauliflower plants in the field **(C)**. Verticillium symptoms on oilseed rape **(D,E)**: Stunted growth and vein clearing in oilseed rape caused by artificial infection of *V. longisporum* **(D)**; Verticillium stem striping in oilseed rape caused by *V. longisporwn*, formation of microsclerotia in the stem cortex beneath the epidermis **(E)**. Pepper plants infected by *V. dahliae* showing wilted leaves **(F)**. Eggplant infected by *V. dahliae* showing chlorosis and necrosis of leaves **(G)**.

Vascular wilts caused by members of the genus *Verticillium* are among the most devastating fungal diseases worldwide. The genus *Verticillium* consists of a relatively small group of soil-borne ascomycete fungi and several of them cause wilt disease on a variety of plant hosts in many parts of the world. Causal agents of Verticillium wilt diseases are globally distributed, most prevalent in temperate and subtropical regions and rare in tropical regions. The consequences of *Verticillium* infection can be far-reaching, leading to huge yield losses (Pegg and Brady, 2002). Currently, 10 species are defined within the *Verticillium* genus (Table 1) of which *Verticillium dahliae* has the broadest host range and infects over 200 plant species (Inderbitzin et al., 2011; Inderbitzin and Subbarao, 2014). *Verticillium* species produce long-lasting resting structures such as microsclerotia, chlamydospores, and resting mycelium in dead or dying plant tissues (Table 1). These resting structures serve as the primary inoculum from which hyphae are formed that directly penetrate the roots of host plants. Subsequently, the fungus reaches the vascular tissue and colonizes the xylem vessels (Puhalla and Bell, 1981; Schnathorst, 1981). Symptoms associated with Verticillium wilt are stunting, chlorosis, wilting, vascular discoloration, and early senescence. However, symptoms can differ considerably between hosts (Fradin and Thomma, 2006) and *Verticillium* species (Figure 1). For example, *Verticillium longisporum* causes wilting in cauliflower but necrosis on oilseed rape (Depotter et al., 2016). In addition, many plants can harbor endophytic populations of *Verticillium* without showing any symptoms and should be considered as “asymptomatic hosts” (Malcolm et al., 2013). Moreover, within the different *Verticillium* species non-pathogenic isolates can be found that do not cause symptoms upon inoculation of host plants. Several of these non-pathogenic *Verticillium* isolates show biocontrol efficacy against Verticillium wilt (Matta and Garibaldi, 1977; Davis et al., 2000; Robinson et al., 2007; Qin et al., 2008; García et al., 2011; França et al., 2013; Zhu et al., 2013; Tyvaert et al., 2014).

**Table 1:** Species within *Verticillium* with their host range and survival structures.

**Species**	**Host range**	**Survival structures**
*Verticillium albo-atrum*	Pestilence wort, Potato, Stinging nettle	microsclerotia, resting mycelium
*Verticillium alfalfae*	Alfalfa	resting mycelium
*Verticillium dahliae*	wide	microsclerotia
*Verticillium isaacii*	Artichoke, Bear's breech, *Brassica* sp., Florist's daisy, Hairy nightshade, Lettuce, Potato, Spinach, Tomato	microsclerotia, resting mycelium, chlamydospores
*Verticillium klebahnii*	Artichoke, Lettuce	microsclerotia, resting mycelium, chlamydospores
*Verticillium longisporum*	Birdrape, Broccoli, Cabbage, Cauliflower, Field mustard, Horseradish, Oilseed rape, Sugar beet, Turnip, Wild radish	microsclerotia
*Verticillium nonalfalfae*	Alfalfa, Cotton, Hop, Petunia, Potato, Spinach, Tomato, Tree of heaven, Wild celery	resting mycelium
*Verticillium nubilum*	Potato	chlamydospores
*Verticillium tricorpus*	Carnation, Larkspur, Lettuce, Potato, Tomato	microsclerotia, resting mycellium, chlamydospores
*Verrticillium zaregamsianum*	Tomato, Potato, Lettuce, Ten weeks stock	microsclerotia, resting mycelium

*Inderbitzin et al., 2011; Inderbitzin and Subbarao, 2014*.

## Current control strategies for verticillium wilt

Control of Verticillium disease is difficult due to the long persistence of the resting structures in the field and the broad host range of some species. Moreover, the pathogen is difficult to manage once it reaches the vascular plant tissue and fungicides appear to be ineffective. Reducing the primary inoculum in the soil has been considered as an important goal and can be accomplished by several management strategies. Chemical fumigants can reduce the inoculum of *Verticillium* in soil, however their use is restricted because of the detrimental effect on the environment. Disease management has been focusing on implementing integrated pest management (IPM). Different IPM strategies to reduce the primary inoculum were recently summarized by the EIP-AGRI focus group of soil-borne diseases (https://ec.europa.eu/eip/agriculture/en/content/focus-groups) and include crop rotation, the use of cover crops, green manures, and organic amendments, and non-chemical soil disinfestation (solarization, soil steaming, anaerobic disinfestation, inundation, and biofumigation). Those management strategies have been implemented into agricultural production and all of them have their specific concerns and limitations.

Another interesting approach is the protection of plants against *Verticillium* by genetic resistance. Resistance has been identified in a limited number of crops and has mainly been studied in tomato, potato and cotton. Grafting on resistant rootstocks is a common strategy to protect vegetables, such as tomato and pepper, against soil-borne pathogens, but is not always effective in controlling Verticillium wilt (Garibaldi et al., 2005; Geboloǧlu et al., 2011). Resistance may break down under high disease pressure, leading to new races of the pathogen or a shift in the pathogen population (Lazarovits and Subbarao, 2009; Colla et al., 2012). For example, Verticillium wilt of tomato was effectively controlled by growing cultivars with resistance against *V. dahliae* race 1 (Schaible et al., 1951). Later on, a shift in the pathogen population occurred and race 2 became dominant (Grogan et al., 1979; Dobinson et al., 1996) for which no resistant cultivars are available.

Another tool for IPM is the use of biological control agents (BCAs), a promising strategy to control soil-borne diseases such as *Verticillium*. Although several microorganisms have shown efficacy against Verticillium wilt, hardly any of them are available as biopesticide against *Verticillium* in Europe (http://ec.europa.eu/food/plant/pesticides/). To increase the use of BCAs in agriculture, some issues for successful practical implementation should be considered in the selection process of potential BCAs and good protocols of use are needed for farmers. In this review, we summarized the research about biocontrol against Verticillium wilt in various crops. The idea was to understand what makes a good BCA against *Verticillium* and how the development of these organisms into an effective biopesticide can be improved.

## Biological control of verticillium wilt

We consulted the Web of Science database until February 28, 2017 using keywords such as “Verticillium,” “Verticillium wilt,” in combination with “biological control,” “biocontrol,” “cross-protection,” and “endophytes” to search for relevant publications. Only studies in which the BCAs or their exudates were tested on plants were considered. Tables 2, 3 give an overview of respectively the bacterial and fungal/oomycete isolates tested against Verticillium wilt. In the table of the fungal and oomycete BCAs all isolates tested against *Verticillium* were included regardless of their effect and their control efficacy is indicated. The taxonomy of the species belonging to the Glomeromycota was adjusted according to the classification proposed by Schüβler and Walker (2010). A different approach was used for bacterial BCAs. Only isolates that could control Verticillium wilt and were identified at least to the genus level were included in the table. For each antagonist, the studied host plant, the effect on growth with and without *Verticillium* and the (possible) mode of action are shown.

**Table 2:** Bacterial isolates with biocontrol activity against *Verticillium* in different host plants.

**Antagonist**	**Host**	Effect on growth^(*)^		**Mode of action**	**References**
		**-Ve**	**+ Ve**		
**GRAM-POSITVE**					
***Arthrobacter***					
*Arthrobacter* sp. FP15	Eggplant			Reduced MS germination, antibiosis (iv), IR	Papasotiriou et al., 2013
***Bacillus***					
*B. amyloliquefaciens* 41B-1	Cotton			Reduced MS germination, antibiosis (iv), IR	Han et al., 2015
*B. amyloliquefaciens* 5-127	Eggplant		+	Antibiosis (iv), mycoparasitism (iv)	Tjamos et al., 2004
	Potato			Antibiosis (iv), mycoparasitism (iv)	Tjamos et al., 2004
*B. amyloliquefaciens* UCMB-5033, UCMB-5036, UCMB-5113	Oilseed rape	+		Antibiosis (iv)	Danielsson et al., 2007
*B. cereus* CH2	Eggplant		+	Reduced spore germination (iv), antibiosis (iv), mycoparasitism (iv)	Li et al., 2008
*B. cereus* AR156	Cotton	+	+	Reduced spore germination (iv)	Yang et al., 2014
*B. pumilus* M1	Potato			Antibiosis (iv)	Uppal et al., 2007, 2008
*B. subtilis* B-26, B-121, B-135, B-136, B-150, B-181	Maple			Antibiosis (iv)	Hall et al., 1986
*B. subtilis* SM21	Cotton	+	+	Reduced spore germination (iv)	Yang et al., 2014
*B. subtilis* YUPP-2	Cotton			Antibiosis (iv)	Yang et al., 2013
*B. subtilis* Jaas ed1	Eggplant			Antibiosis (iv)	Lin et al., 2009
*B. subtilis* DF14	Cotton				Luo et al., 2010
*B. subtilis* TS06	Strawberry	+		Reduced spore germination, antibiosis (iv)	Zhang Y. et al., 2012
*B. subtilis* HJ5	Cotton			Antibiosis (iv), competition	Li et al., 2013
*B. vallismortis* HJ-5	Cotton		+		Zhang G. et al., 2012
*Bacillus* sp. K-160	Eggplant		+	Antibiosis (iv), mycoparasitism (iv)	Tjamos et al., 2004
***Paenibacillus***					
*P. alvei* K-165	Eggplant		+	Reduced MS germination, antibiosis (iv), mycoparasitism (iv), IR	Tjamos et al., 2004; Antonopoulos et al., 2008; Markakis et al., 2008; Angelopoulou et al., 2014
	Potato			Antibiosis (iv), mycoparasitism (iv)	Tjamos et al., 2004
	Arabidopsis			IR	Tjamos et al., 2005; Gkizi et al., 2016
	Olive				Markakis et al., 2016
*P. polymyxa* YUPP-8	Cotton			Antibiosis (iv)	Yang et al., 2013
*P. xylanilyticus* YUPP-1	Cotton			Antibiosis (iv)	Yang et al., 2013
***Streptomyces***					
*S. albidoflavus* S1	Strawberry			Antibiosis (iv), mycoparasitism (iv)	Berg et al., 2000
*S. albidoflavus* 1W1	Strawberry		+		Berg et al., 2001
*S. cyaneofuscatus* ZY-153	Cotton	+	0	Antibiosis (iv), mycoparasitism (iv), IR	Xue et al., 2013, 2016
*S. diastatochromogenes* S9	Strawberry			Antibiosis (iv), mycoparasitism (iv)	Berg et al., 2000
*S. flavotricini* Z-13	Cotton	+	0	Antibiosis (iv), mycoparasitism (iv), IR	Xue et al., 2013, 2016
*S. kanamyceticu* B-49	Cotton	+	0	Antibiosis (iv), mycoparasitism (iv), IR	Xue et al., 2013, 2016
*S. lividans* 66	Arabidopsis	+	+	Reduced spore germination, reduced MS formation, antibiosis (iv)	Meschke and Schrempf, 2010; Meschke et al., 2012
*S. lydicus* WYEC108 (wood chip-PAM cores)	Potato		+	Competition	Entry et al., 2000
*S. rimosus* 7W1	Strawberry		0		Berg et al., 2001
*S. rochei* X-4	Cotton	+	+	Antibiosis (iv), mycoparasitism (iv), IR	Xue et al., 2013, 2016
*Streptomyces* sp. DHV3-2	Tomato	+	+	Antibiosis (iv)	Cao et al., 2016
**GRAM-NEGATIVE**					
***Acetobacter***					
*A. aceti* VIN02	Olive			Reduced MS germination, mycoparasitism (iv)	Varo et al., 2016b
***Enterobacter***					
*Enterobacter* sp. AS09	Oilseed rape		+	Antibiosis (iv), mycoparasitism (iv)	Alström, 2001
*Enterobacter* sp. HA02	Cotton	+	+	Mycoparasitism (iv)	Li et al., 2010, 2012
***Pseudomonas***					
*P. chlororaphis* K15	Strawberry	+	+	Antibiosis (iv), mycoparasitism (iv)	Berg et al., 2001
*P. chlororaphis* MA342	Oilseed rape	+	+		Abuamsha et al., 2011
*P. fluorescens* M-4	Potato	0	+	Competition	Leben et al., 1987
*P. fluorescens* P6, P10	Strawberry			Antibiosis (iv), mycoparasitism (iv)	Berg et al., 2000
*P. fluorescens* B6, B41	Eggplant			Antibiosis (iv)	Malandraki et al., 2008
*P. fluorescens* DF37	Potato			Antibiosis (iv)	Uppal et al., 2007, 2008
*P. fluorescens* PICF4, PICF6, PICF8	Olive	0	+	Antibiosis (iv)	Mercado-Blanco et al., 2004; Varo et al., 2016b
*P. fluorescens* PICF7	Olive	0	+	Competition, IR	Mercado-Blanco et al., 2004; Prieto et al., 2009; Schilirò et al., 2012; Gómez-Lama Cabanás et al., 2014; Maldonado-González et al., 2015b
	Arabidopsis				Maldonado-González et al., 2015a
*P. putida* B E2	Strawberry	+	+	Antibiosis (iv), mycoparasitism (iv)	Berg et al., 2001
*P. putida* PICP2	Olive	0	0	Antibiosis (iv)	Mercado-Blanco et al., 2004
*P. putida* PICP5	Olive	0	+	Antibiosis (iv)	Mercado-Blanco et al., 2004
*Pseudomonas* sp. FP22, FP23, FP30, FP35	Cotton	+	+	Antibiosis (iv)	Erdogan and Benlioglu, 2010
***Serratia***					
*S. plymuthica* HRO-C48	Strawberry	+	+	Mycoparasitism (iv)	Kalbe et al., 1996; Kurze et al., 2001
	Cotton		+	Mycoparasitism (iv)	Kalbe et al., 1996; Erdogan and Benlioglu, 2010
	Oilseed rape	+	+	Mycoparasitism (iv)	Kalbe et al., 1996; Müller and Berg, 2008; Abuamsha et al., 2011
*Serratia* sp. XY21	Cotton	+	+	Reduced spore germination (iv)	Yang et al., 2014
***Stenotrophomonas***					
*S. maltophilia* (isolate 1)	Oilseed rape			Antibiosis (iv), mycoparasitism (iv)	Berg et al., 1996
*Stenotrophomonas* AS10	Oilseed rape		+	Antibiosis (iv), mycoparasitism (iv)	Alström, 2001

* *Plant growth promotion with or without Verticillium infection is represented by “+” and a negative effect on the growth by “−”. No effect on the growth is indicated by “0”. iv, in vitro; IR, Induced Resistance; PAM: polyacrylamide*.

**Table 3:** Fungal and oomycete isolates with potential biocontrol activity against *Verticillium* in different host plants.

**Antagonist**	**Host**	Control efficiency(^*^)	Effect on growth(^**^)		**Mode of action**	**References**
		**Disease**	**−Ve**	**+Ve**		
**OOMYCOTA**						
***Pythium***						
*P. oligandrum* (Polyversum®)	Pepper	+		+		Rekanovic et al., 2007
	Tomato			0		Giotis et al., 2009
*P. oligandrum (mixture of 5 isolates)*	Pepper		+	+	Reduced MS production, mycoparasitism (iv)	Al-Rawahi and Hancock, 1998
**ASCOMYCOTA**						
***Acremonium***						
*Acremonium* sp. CEF-193	Cotton	+	0	0	Antibiosis (iv)	Li et al., 2014; Yuan et al., 2017
***Alternaria***						
*Alternaria* sp. RF4	Oilseed rape			0	Mycoparasitism (iv)	Alström, 2000
***Aspergillus***						
*A. alutaceus*	Eggplant	0				Marois et al., 1982
***Aureobasidium***						
*A. pullulans* AP06	Olive	0			Antibiosis (iv)	Varo et al., 2016b
***Blastobotrys***						
*Blastobotrys* sp. FP12	Eggplant	+			Reduced MS germination, antibiosis (iv), IR	Papasotiriou et al., 2013
***Chaetomium***						
*C. globosum* B221, A354, *Chaetomium* sp.	Cotton	+	+		Antibiosis (iv), mycoparasitsm (iv)	Zheng et al., 2011
***Fusarium***						
*F. culmorum*	Tomato	+	0	+	Antibiosis (iv)	Dutta, 1981
*F. lateritium* BAFC2317 (ex)	Tomato		0	+	Antibiosis (iv), DAMP release	García et al., 2011
*F. moniliforme* FM01	Olive	0				Varo et al., 2016a
*F. moniliforme* FM02	Olive	+			Antibiosis (iv), IR	Varo et al., 2016b
*F. oxysporum* FO03, FO04	Olive	+			Reduced MS germination, antibiosis (iv)	Varo et al., 2016b
*F. oxysporum* FO12	Olive	+			Reduced MS germination, antibiosis (iv), IR	Varo et al., 2016a,b
*F. oxysporum* CanR-46	Cotton	+			Reduced germination of inoculum (VOCs, iv), antibiosis (VOCs, iv)	Zhang et al., 2015
*F. oxysporum* f. sp. *lycopersici* CECT 2715	Pepper	+	0	+	IR	Díaz et al., 2005
*F. oxysporum* f. sp. *lycopersici, F. oxysporum* f. sp. *dianthi*	Tomato	+				Matta and Garibaldi, 1977
*F. oxysporum* F2	Eggplant	+			Competition, IR	Malandraki et al., 2008; Pantelides et al., 2009; Gizi et al., 2011, Angelopoulou et al., 2014
*F. oxysporum* F4	Eggplant	+				Malandraki et al., 2008
*F. oxysporum* Fo47	Pepper	+	0	+	IR	Veloso and Díaz, 2012
	Olive	0				Varo et al., 2016b
*F. oxysporum* Fo47b10	Eggplant	^(1)^			mycoparasitism	Nagtzaam et al., 1998
	Potato	^(2)^				Nagtzaam et al., 1998
*F. oxysporum* By125, Ja127, *F. equiseti* By222, *F. solani* Bx 215	Cotton	+	+		Antibiosis (iv), mycoparasitism (iv)	Zheng et al., 2011
*Fusarium* sp. Bx144	Cotton	+	0		Mycoparasitism (iv)	Zheng et al., 2011
*Fusarium* sp. MTB1, MNS1, MNB3	Eggplant	+				Narisawa et al., 2002
*Fusarium* sp. RF6	Oilseed rape			0	Parasitism (iv)	Alström, 2000
***Gibellulopsis***						
*G. nigrescens* CVn-WHg	Cotton	+	0	+		Zhu et al., 2013
*G. nigrescens* (formerly *V. nigrescens*)	Peppermint, spearmint	+				Melouk and Horner, 1975
*G. nigrescens* (formerly *V. nigrescens*)	Cotton	+	0	+		Vagelas and Leontopoulos, 2015
***Gliocladium***						
*G. roseum* GR01	Olive	0				Varo et al., 2016a
*G. roseum* GR02	Olive	0			Reduced MS germination, antibiosis (iv)	Varo et al., 2016b
*Gliocladium* sp. RF12	Oilseed rape	+		+	Antibiosis (iv), mycoparasitism (iv)	Alström, 2000
*Gliocladium* sp. RF15	Oilseed rape			0	Mycoparasitism (iv)	Alström, 2000
*Gliocladium* sp.	Tomato	+	+	+	Antibiosis (iv), mycoparasitism (iv)	Dutta, 1981
***Heteroconium***						
*H. chaetospira* H4007	Chinese cabbage	+		0		Narisawa et al., 2000, 2004
*H. chaetospira* MNB4	Eggplant	+				Narisawa et al., 2002
***Leptosphaeria***						
*Leptosphaeria* sp. CEF-714	Cotton	+	0	0	Antibiosis (iv)	Li et al., 2014; Yuan et al., 2017
***Microsphaeropsis***						
*M. ochracea*	Oilseed rape	0			Reduced MS germination, mycoparasitism	Stadler and von Tiedemann, 2014
***Muscodor***						
*M. albus* 620, *M. roseus* A3-5	Eggplant	+			Reduced MS germination	Stinson et al., 2003
**Mycelium radicis atrovirens (MRA)**						
MRA MTJ1, MRA MIB3, MRA MNB9	Eggplant	+				Narisawa et al., 2002
***Myrothecium***						
*M. roridum* A243	Cotton	+	+		Mycoparasitism (iv)	Zheng et al., 2011
***Nectria***						
*N. haematococca* Bx247	Cotton	+	+		Mycoparasitism (iv)	Zheng et al., 2011
**Non sporulating fungus with white mycelium**						
SWM MHB2	Eggplant	+				Narisawa et al., 2002
***Paecilomyces***						
*P. lilacinus*	Eggplant	+				Marois et al., 1982
***Penicillium***						
*P. chrysogenum* (dm)	Cotton	+			IR	Dong et al., 2003, 2006
*P. chrysogenum*	Cotton	+				Zhang et al., 2011
*P. chrysogenum* EEZ10 (ex)	Tomato		0	+	Antibiosis (iv), DAMP release	García et al., 2011
*P. chrysogenum, P. vermiculatum, Penicillium* sp.	Tomato	+	+	+	Antibiosis (iv), mycoparasitism (iv)	Dutta, 1981
*P. oxalicum* PO212	Tomato	+				Larena et al., 2003; Sabuquillo et al., 2005, 2006
*P. simplicissimum* CEF-818	Cotton	+	0	+	Antibiosis (iv), IR	Li et al., 2014; Yuan et al., 2017
*Penicillium* sp. MNT8	Eggplant	+				Narisawa et al., 2002
***Phialocephala***						
*P. fortinii* J2PC2, LtPE2	Chinese cabbage	−		0		Narisawa et al., 2004
*P. fortinii* MNJ1	Eggplant	+				Narisawa et al., 2002
***Phoma***						
*Phoma* sp. PH01	Olive	+			Reduced MS germination, antibiosis (iv), IR	Varo et al., 2016b
*Phoma* sp. PH02	Olive	+				Varo et al., 2016a
***Phomopsis***						
*Phomopsis* sp. By231	Cotton	+	+		Antibiosis (iv), mycoparasitism (iv)	Zheng et al., 2011
*Phomopsis* sp. By254	Cotton	+	0/−		Antibiosis (iv)	Zheng et al., 2011
***Talaromyces***						
*T. flavus*	Eggplant	+				Marois et al., 1982
*T. flavus* Po-V-48, Po-V-49, Po-V-50, Po-V-51, Po-V-52	Potato	+			Antibiosis (iv)	Naraghi et al., 2010b
*T. flavus* Cu-V-55, Cu-V-57, Cu-V-58, Cu-V-59, Cu-V-60	Cucumber	+			Antibiosis (iv)	Naraghi et al., 2010a
*T. flavus*	Tomato, cucumber	0				Zeise and Kersten, 2000
	Oilseed rape	+				
	Strawberry	+				
*T. flavus Tf-1*	Hop	+	0	+		Solarska et al., 2000
*T. flavus TN11 and TN41*	Potato	^(2)^			Reduced MS germination	Nagtzaam et al., 1998
	Eggplant	^(7)^			Mycoparasitism	
*T. flavus* CEF-642	Cotton	+	0	0	Antibiosis (iv)	Li et al., 2014; Yuan et al., 2017
***Trichoderma***						
*T. asperellum* B35	Pepper	+				Ślusarski and Pietr, 2009
*T. asperellum* B35	Hop		+	0		Solarska et al., 2000
*T. asperellum* T-34	Strawberry	^(3)^				Martinez et al., 2009
*T. asperellum* Bt3	Olive	+	0	0	Antibiosis (iv)	Carrero-Carrón et al., 2016
*T. asperellum* T25	Olive	+	+	+	Antibiosis (iv)	Carrero-Carrón et al., 2016
*T. asperellum* + *T. gamsii* (BIOTEN®)	Olive	+			Reduced MS germination, antibiosis (iv)	Varo et al., 2016b
*T. harzianum* T-22 (GTG II®)	Spinach	^(4)^				Cummings et al., 2009
*T. harzianum* T-22 (Planter Box Biological Fungicide®)	Spinach	^(5)^				Cummings et al., 2009
*T. harzianum* T-35	Potato	+				Ordentlich et al., 1990
*T. harzianum*	Eggplant	0				Marois et al., 1982
*T. harzianum* (promot®)	Strawberry	0^(6)^	0	0^(6)^		Weissinger et al., 2009
*T. harzianum* T3, T94, T106, T108, T120, *T. viride* T9, T46, T67, T107, T117	Eggplant	+		+	Antibiosis (iv), mycoparasitism (iv)	D'Ercole et al., 2000
*T. harzianum* TU63, TU68, TU72, TU74, TU75, TU79, TU80	Strawberry	+			Antibiosis (iv)	Mirmajlessi et al., 2016
*T. viride*	Tomato	+	+	+	Antibiosis (iv), mycoparasitism (iv)	Dutta, 1981
*T. viride*	Eggplant	+				Marois et al., 1982
*T. virens* (formerly *Gliocladium virens*)	Eggplant	0				Marois et al., 1982
*Trichoderma* sp. MNS11	Eggplant	+				Narisawa et al., 2002
*Trichoderma* sp. RF14, RF16	Oilseed rape			0	Mycoparasitism (iv)	Alström, 2000
***Verticillium***						
*V. albo-atrum* SS-4	Cotton	+				Schnathorst and Mathre, 1966
*V. albo-atrum* T-1	Tomato	0				Schnathorst and Mathre, 1966
*V. albo-atrum, V. tricorpus*	Potato	+				Robinson et al., 2007
*V. alfalfae* (formerly *V. albo-atrum*), *V. tricorpus*	Tomato	+				Matta and Garibaldi, 1977
*V. dahliae* Dvd-E6	Tomato	+	+	+	IR	Shittu et al., 2009
*V. dahliae 2379* (ex)	Tomato		+	+	DAMP release	García et al., 2011
*V. dahliae* (CVd-WHw)	Cotton	+	+	+		Zhu et al., 2013
*V. isaacii* Ls. 432, Ls. 443 (formerly *V. tricorpus*),	Lettuce	+	0	0		Qin et al., 2008
*V. isaacii* Ls. 441, Ls 442, Ls. 183 (formerly *V. tricorpus*)	Lettuce	+				Qin et al., 2008
*V. isaacii*	Cauliflower	+				França et al., 2013
*V. isaacii* Vt305	Cauliflower	+				Tyvaert et al., 2014
*V. tricorpus* V-17, V-28, V-31	Potato	+				Davis et al., 2000
**BASIDIOMYCOTA**						
***Coriolopsis***						
*C. rigida* CECT20449 (ex)	Tomato		0	+	Antibiosis (iv), DAMP release	García et al., 2011
**Dark septate endophytes**						
isolate *LtVB3*	Chinese cabbage	+		+		Narisawa et al., 2004
DSE48	Tomato	0	0	0		Andrade-Linares et al., 2011
DSE49	Tomato	+	+	+		Andrade-Linares et al., 2011
*Leptodontidium orchidicola*	Tomato	+	0	+		Andrade-Linares et al., 2011
***Piriformospora***						
*P. indica*	Tomato	+	+	+		Fakhro et al., 2010
***Trametes***						
*T. versicolor* A136 (ex)	Tomato		0	+	Antibiosis (iv), DAMP release	García et al., 2011
**GLOMEROMYCOTA**						
***Claroideoglomus***						
*C. claroideum* (formerly *G. claroideum*)	Olive	0	+			Porras-Soriano et al., 2006
*C. etunicatum* (formerly *G. etunicatum*)	Eggplant	+	+		IR	Matsubara et al., 1995
***Funneliformis***						
*F. mosseae* (formerly *G. mosseae*)	Tomato, pepper	+	0	0		Demir et al., 2015
*F. mosseae* (formerly *G. mosseae*)	Tomato, eggplant		+	+		Karagiannidis et al., 2002
*F. mosseae* (formerly *G. mosseae*)	Alfalfa	+	+			Hwang et al., 1992
*F. mosseae* (formerly *G. mosseae*)	Pepper	0	0	0		Garmendia et al., 2004c
*F. mosseae* (formerly *G. mosseae*)	Olive	0	+			Porras-Soriano et al., 2006
*F. mosseae* (formerly *G. mosseae*)	Cotton	+	+	+		Liu, 1995
*F. mosseae* (formerly *G. mosseae*) *+ F. caledonium* (*G. caledonium*)	Tomato	0	0	0		Baath and Hayman, 1983
***Gigaspora***						
*G. margarita*	Eggplant	+	+		IR	Matsubara et al., 1995
***Glomus***						
*G. deserticola*	Pepper	+	0	0	IR	Garmendia et al., 2004a,b,c; Garmendia et al., 2006
*G. hoi*	Cotton	+	0	0		Liu, 1995
*G. versiforme*	Cotton	+	+	+		Liu, 1995
*G. versiforme*	Cotton	+		+		Zhang G. et al., 2012
*Glomus* sp.	Alfalfa	+	+			Hwang et al., 1992
***Rhizophagus***						
*R. fasciculatus* (formerly *G. fasciculatus)*	Alfalfa	+	+			Hwang et al., 1992
*R. fasciculatus* (formerly *G. fasciculatus)*	Cotton	0	+	0		Davis et al., 1979
*R. intraradices* (formerly *G. intraradices*)	Eggplant	+	0	0		Demir et al., 2015
*R. intraradices* (formerly *G. intraradices*)	Pepper	0	0	0		Garmendia et al., 2004c
*R. intraradices* (formerly *G. intraradices*)	Olive	0	+			Porras-Soriano et al., 2006
*R. intraradices* (formerly *G. intraradices*)	Olive	0	+			Kapulnik et al., 2010
***Sclerocystis***						
*S. sinuosa*	Cotton		0	0		Liu, 1995
**ZYGOMYCOTA**						
*Mortierella* sp. RF1, RF2	Oilseed rape			0	Mycoparasitism (iv)	Alström, 2000

* *A reduction or increase of disease incidence or/and severity is indicated by respectively “+” and “−”. No effect on the disease is indicated by “0”. Isolates with biocontrol activity are also marked in green*.

** *Plant growth promotion with or without Verticillium infection is represented by “+” and a negative effect on the growth by “−”. No effect on the growth is indicated by “0”*.

(1) Reduced Verticillium colonization of the roots but not of the stem;

(2) *No reduced Verticillium colonization*;

(3) Trichoderma population was negatively affected by V. dahliae;

(4) *Reduced % of Verticillium infested seeds*;

(5) *No reduced % of Verticillium infested seeds*;

(6) *No Verticillium symptoms developed during experiments*;

(7) *Reduced Verticillium colonization of the roots and stem. ex, exudates of the isolate were used to apply to the plants; dm, dry mycelium of the isolate was applied to the plants; iv, in vitro; IR, Induced Resistance*.

### Studied host plants

Pathogenic *Verticillium* species affect a wide variety of plants and in particular *V. dahliae* has a broad host range, including important agricultural crops, woody species, and ornamentals (Pegg and Brady, 2002; Inderbitzin and Subbarao, 2014). Biological control of Verticillium wilt, however, has only been investigated for a few host plants. Studies with bacterial isolates were performed on nine different host plants belonging to six plant families, while studies with fungal and oomycete isolates were performed on 17 different host plants of 11 plant families (Table 2, 3). Most biocontrol studies were focused on plants of the *Solanaceae, Malvaceae*, and *Brassicaceae*. In these families eggplant, cotton and oilseed rape were the most studied crops. Studies on economically important woody species and ornamentals are limited to olive and *Acer* species. This may indicate that isolates controlling Verticillium wilt of woody plants are hard to find. A more likely explanation is that investigating biocontrol in these plants is time-consuming and labor-intensive. Moreover, except for maple and olive, *Verticillium* isolates of woody plants have not been studied extensively and information about their pathogenicity and genetic diversity is limited (Pegg and Brady, 2002; Chandelier et al., 2003; López-Escudero and Mercado-Blanco, 2011).

It should be noted that many of the potential BCAs were tested only once. The reasons can be that those isolates (1) were studied for scientific purposes only, (2) were not considered for further research or (3) insufficient control was established.

### Bacterial biocontrol agents

The potential of bacterial endophytes as biocontrol agents of vascular wilts has recently been reviewed by Eljounaidi et al. (2016). In our study, we specifically focused on Verticillium wilt and included also non-endophytic bacterial BCAs. We divided bacterial biocontrol agents in Gram-positive and Gram-negative bacteria and further arranged them according to their genus (Table 2). Within the Gram-positive bacteria, strains belonging to the genera *Arthrobacter, Bacillus, Paenibacillus*, and *Streptomyces* have been studied. *Bacillus* species comprise the largest group within the Gram-positive bacteria, followed by *Streptomyces* and *Paenibacillus* species. The Gram-negative strains belong to the genera *Acetobacter, Enterobacter, Pseudomonas, Serratia*, and *Stenotrophomonas*, with *Pseudomonas* as the largest pool of potential BCAs of *Verticillium*.

The genus *Bacillus* is well-explored in the search of BCAs to control Verticillium wilt. Over two third of the *Bacillus* strains tested belong to the species *Bacillus amyloliquefaciens* and *Bacillus subtilis*. Remarkably, only the *Bacillus* strain *B. amyloliquefaciens* 5-127, isolated from tomato roots, was tested on different host plants. *B. amyloliquefaciens* 5–127 reduced the percentage of diseased leaves by 40–70% in eggplants challenged with *V. dahliae* in the greenhouse and could reduce disease incidence with more than 50% in a field experiment with potato (Tjamos et al., 2004). In one of the few studies regarding biological control of Verticillium wilt in trees, several *B. subtilis* isolates were tested in the greenhouse against *V. dahliae* in maple tree. These isolates were obtained from healthy maple stem tissue and decreased disease incidence of *V. dahliae* in maple trees by 34–51% (Hall et al., 1986). *Bacillus* strains were also reported to protect cotton, strawberry and oilseed rape against Verticillium wilt (Table 2).

*Paenibacillus* isolates have recently gained interest as promising BCAs of plant diseases (Lal and Tabacchioni, 2009; Rybakova et al., 2016). *Paenibacillus alvei* K-165 was isolated from tomato root tips grown in solarized soil (Tjamos et al., 2004) and its biocontrol activity against *V. dahliae* in eggplant has repeatedly been shown in greenhouse experiments (Tjamos et al., 2004; Antonopoulos et al., 2008; Markakis et al., 2008; Angelopoulou et al., 2014). This strain also reduced the disease incidence in potato under field conditions and suppressed Verticillium wilt of olive tree under both greenhouse and field conditions (Tjamos et al., 2004; Markakis et al., 2016). In cotton, application of the *Paenibacillus* isolates *P. xylanilyticus* YUPP-1 and *Paenibacillus polymyxa* YUPP-8 resulted in a lower disease incidence and decreased severity of *Verticillium* (Yang et al., 2013).

Various species of *Streptomyces* have been studied in relation to their biological control effect against *Verticillium*. Xue et al. (2013) selected four *Streptomyces* strains isolated from the rhizosphere of different crops and evaluated their antagonistic potential against *V. dahliae* in cotton. Under greenhouse conditions the biocontrol efficacy ranged between 19 and 66%, while in field conditions the biocontrol efficacies of the four *Streptomyces* isolates were slightly lower and ranged between 14 and 51% depending on the application method. Co-inoculation of *Arabidopsis thaliana* seeds with *V. dahliae* and *Streptomyces lividans* 66 led to a strong suppression of the fungus within soil, which resulted in a strong reduction of Verticillium-induced disease symptoms (Meschke and Schrempf, 2010). In potato, tomato and strawberry, *Streptomyces* species reduced the disease incidence and/or severity in greenhouse experiments (Berg et al., 2000, 2001; Entry et al., 2000; Cao et al., 2016). However, the biofungicide Mycostop® based on *S. griseovirides* K61 did not offer significant protection against *V. dahliae* in tomato (Minuto et al., 2006).

*Pseudomonas* spp. have been extensively studied as BCA of different pathogens including *Verticillium*. Most of the tested potential biocontrol strains belong to the fluorescent *Pseudomonas* group. Root treatment of olive plants with root-associated fluorescent pseudomonads during nursery propagation could suppress Verticillium wilt in olive caused by defoliating *V. dahliae* (Mercado-Blanco et al., 2004; Prieto et al., 2009). Other isolates of the fluorescent *Pseudomonas* group can be protective against *V. dahliae* in crops such as potato, strawberry, and eggplant (Leben et al., 1987; Berg et al., 2000, 2001; Malandraki et al., 2008; Uppal et al., 2008). Seed treatment with *P. chlororaphis* strain MA 342, the active organism in the biopesticides Cedomon® and Cerall® (BioAgri AB, Uppsala, Sweden), resulted in a lower infection of oilseed rape with *V. longisporum* (Abuamsha et al., 2011). The study of Erdogan and Benlioglu (2010) indicated that the *Pseudomonas* strains FP22, FP23, FP30 and FP35 are good biocontrol candidates against Verticillium wilt of cotton and moreover can improve the growth parameters in cotton fields.

Isolates of the Gram-negative genus *Serratia* have frequently been found associated with plant roots and possess antifungal properties (Grimont and Grimont, 1992; Kalbe et al., 1996). The biocontrol strain *Serratia plymuthica* HRO-C48 successfully controlled Verticillium wilt in strawberry fields (Kurze et al., 2001). Furthermore, treating the seeds of oilseed rape with *S. plymuthica* HRO-C48 via bio-priming, pelleting or seed coating suppressed Verticillium wilt in oilseed rape plants (Müller and Berg, 2008). Seed treatment with *S. plymuthica* HRO-C48 could also protect cotton plants against Verticillium wilt (Erdogan and Benlioglu, 2010).

The application of specific isolates belonging to the genera *Arthrobacter, Acetobacter, Enterobacter*, and *Stenotrophomonas* resulted in protection of eggplant, olive, cotton and oilseed rape against Verticillium wilt (Berg et al., 1996; Alström, 2001; Li et al., 2012; Papasotiriou et al., 2013; Varo et al., 2016b).

### Fungal and oomycete biocontrol agents

Fungal and oomycete isolates tested as BCA against *Verticillium* are listed in Table 3. The majority of isolates belong to the Ascomycota and a minor fraction of the isolates belong to the Basidiomycota and Glomeromycota. Only one Oomycete, *Pythium oligandrum*, has been investigated. Studies with *Trichoderma, Fusarium*, and *Verticillium* isolates as potential biocontrol agent were the most prevalent. Isolates of *Talaromyces, Funneliformis, Rhizophagus, Glomus*, and *Penicillium* have been studied more than three times. Isolates of other species were less frequently considered as BCA.

*Talaromyces flavus* reduced Verticillium disease of eggplant and potato with more than 75% in naturally infested soils (Marois et al., 1982; Naraghi et al., 2010b). Different formulations of *T. flavus* were tested (Nagtzaam et al., 1998; Zeise and Kersten, 2000), but up to date none of them have been registered in the European Union (http://ec.europa.eu/food/plant/pesticides).

Control of *Verticillium* by arbuscular mycorrhizal fungi (AMF) of the Glomeromycota is variable. Twelve of the tested strains could effectively protect plants against the disease with a maximum reduction of the disease incidence with 65%, while some of the AMF even worsened the disease (Davis et al., 1979; Porras-Soriano et al., 2006). Interestingly, *Glomus deserticola* influenced the plant phenology of pepper plants which contributed to more resistant or tolerant plants to pathogen attack (Garmendia et al., 2004c).

Some *Penicillium* isolates or their exudates or dry mycelium were tested for potential biocontrol. In cotton, the application of dry mycelium resulted in a control efficacy of 27–50% depending on the applied dose (Dong et al., 2006). Exudates of *Penicillium chrysogenum* EEZ10 decreased the negative effect of *Verticillium* on the plant growth of tomato (García et al., 2011). The formulation of *Penicillium oxalicum* PO-212 spores influenced the efficacy: mixing the conidia with the substrate gave better control compared to applying the conidial suspension immediately to the seedbed (Larena et al., 2003).

A lot of isolates belonging to *Trichoderma* have been evaluated for their capacity to control Verticillium wilt with variable successes. Ten *Trichoderma* isolates were tested by D'Ercole et al. (2000) and *Trichoderma viride* T46 and T117 resulted in the best protection with a reduction of the disease incidence of 30% in eggplant. Three strains reduced the disease with more than 80% in tomato, eggplant and pepper (Dutta, 1981; Narisawa et al., 2002; Ślusarski and Pietr, 2009). In the case of respectively *Trichoderma asperellum* B35 and *Trichoderma harzianum* T-35, the efficacy of control depended on several factors such as the field location of the experiments and the type of formulation (Ordentlich et al., 1990; Ślusarski and Pietr, 2009). In olive, *T. asperellum* isolates T25 and Bt3 and application of BIOTEN® (*T. asperellum* + *T. gamsii*) reduced the disease severity of Verticillium wilt but not the incidence (Carrero-Carrón et al., 2016; Varo et al., 2016b).

Recently, *Fusarium oxysporum* isolates have gained interest as BCA against Verticillium wilt. *F. oxysporum* is also a soil-borne fungi and able to colonize and penetrate the roots of host plants. *F. oxysporum* F2 has been extensively studied for its biocontrol capacity on eggplant and reduced disease severity and colonization by *V. dahliae* (Malandraki et al., 2008; Pantelides et al., 2009; Gizi et al., 2011; Angelopoulou et al., 2014). The strain was applied by seed treatment or amendment to the transplant soil plug. This last strategy gave the best results with a dose dependent response. Pepper and olive plants treated with *F. oxysporum* isolate Fo47 exhibited reduced symptoms (Veloso and Díaz, 2012; Varo et al., 2016b). In the case of olive, the *F. oxysporum* isolates FO04 and FO12 showed stronger biocontrol activity against Verticillium wilt than isolate Fo47 (Varo et al., 2016a,b). In cotton, *F. oxysporum* By125 and *F. oxysporum* CanR-46 reduced disease severity with respectively 69 and 92% (Zheng et al., 2011; Zhang et al., 2015). Applying exudates of *Fusarium lateritium* to tomato roots decreased the negative effect of *V. dahliae* on the growth of the plants (García et al., 2011).

Different isolates belonging to *V. dahliae, Verticillium albo-atrum, Verticillium isaacii, Verticillium tricorpus*, and *Gibellulopsis nigrescens* (formerly *Verticillium nigrescens*) protected plants against a virulent relative of *Verticillium* spp. The isolate *V. dahliae* Dvd-E6 was non-pathogenic on tomato and conferred protection to tomato plants challenged with the pathogen *V. dahliae*. The order of inoculation of both isolates influenced the level of protection (Shittu et al., 2009). Applying exudates of *V. dahliae* 2379 to tomato roots decreased plant growth reduction by a pathogenic *V. dahliae* isolate (García et al., 2011). In cotton, Verticillium wilt was reduced by *V. albo-atrum* SS-4 and *G. nigrescens* (Schnathorst and Mathre, 1966; Zhu et al., 2013; Vagelas and Leontopoulos, 2015). In all those studies, pre-inoculation of the protective isolate appeared to be more robust at reducing Verticillium symptoms relative to co-inoculation. The amount of inoculum applied also played a role for the level of protection by *V. albo-atrum* SS-4 (Schnathorst and Mathre, 1966). Two isolates, *V. dahliae* Dvd-E6 and *V. albo-atrum* SS-4, were able to reduce symptom development in respectively tomato and cotton, but were pathogenic on other host plants (Schnathorst and Mathre, 1966; Dobinson et al., 1998).

*V. tricorpus* and *V. isaacii* (formerly *V. tricorpus*) were both associated with soil suppressiveness of Verticillium wilt in respectively potato and cauliflower fields (Davis et al., 2000; França et al., 2013). *V. isaacii* Vt305, an isolate obtained from the suppressive cauliflower field, has shown to be able to reduce symptom development and colonization by *V. longisporum* of cauliflower (Tyvaert et al., 2014). The control was dependent on the applied dose of both the pathogen and the BCA. Robinson et al. (2007) found that *V. tricorpus* reduced Verticillium disease of potato with 74% in a field experiment and pre-inoculation resulted in the best protection. In the same study, protection by a *V. albo-atrum* isolate was comparable. Also the colonization of the different potato tissues by the pathogenic *V. albo-atrum* isolate was remarkably reduced by pre-inoculation with *V. tricorpus* or *V. albo-atrum*. Several *V. isaacii* isolates reduced Verticillium wilt of lettuce and pretreatment appeared to provide better protection than co-inoculation (Qin et al., 2008).

### Modes of action of the studied BCAs

**Figure 2:**
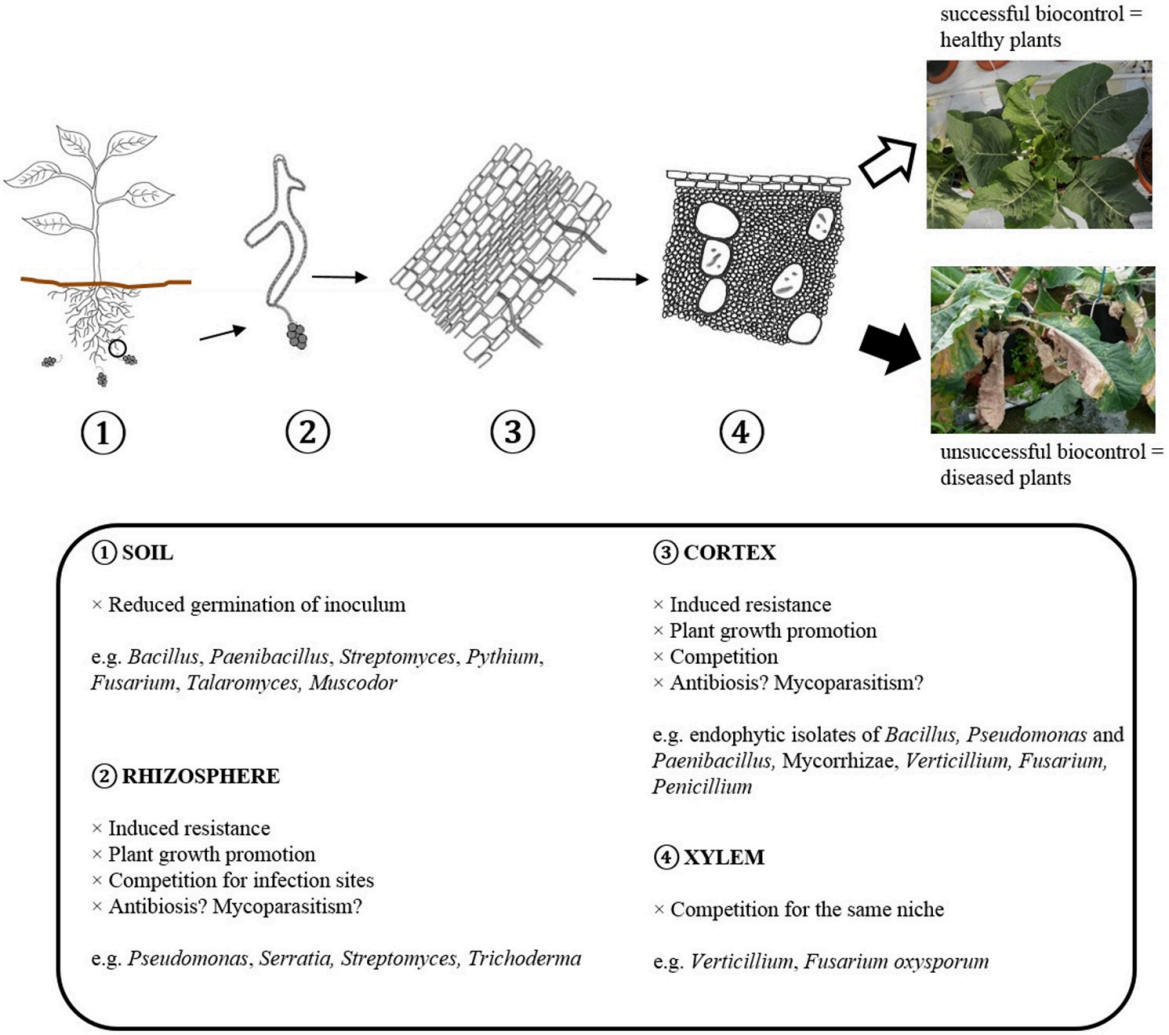
Scheme showing how BCAs can interfere with different steps in the infection cycle of *Verticillium*.

Several modes of action are known to be involved in biological disease control, but the underlying mechanisms of specific interactions with pathogenic *Verticillium* isolates are often unknown. The modes of action reported for the different genera of antagonists against Verticillium wilt are shown in Table 4. Figure 2 shows how BCAs can interfere with different steps in the infection cycle of *Verticillium*. Direct microbial antagonism involves parasitism of the fungus and its surviving structures, competition for nutrients and infection sites or antibiosis. This leads to less inoculum present in the rhizosphere or a lower infection potential of the pathogen. Indirect mechanisms include plant growth promotion and induced resistance. Several bacterial and fungal BCAs promote plant growth and in this way the deleterious effects of Verticillium wilt are reduced. Induced resistance can also contribute to the protection against Verticillium wilt, particularly if this process is initiated in the root tissue which is primarily colonized by the pathogen. Often, several mechanisms are expressed by a single biocontrol agent and one mode of action does not necessarily excludes another.

**Table 4:** Mode of action of selected biocontrol agents against Verticillium wilt.

**Genus antagonist**	**Reduced germination of inoculum**	**Plant growth promotion**	**Competition for infection sites/space/nutrients**	**Induced resistance**	**Antibiosis *in vitro***	**Mycoparasitism *in vitro***
**BACTERIA**						
*Bacillus*	x (iturins)	x	x	x (iturins)	x	x
*Paenibacillus*	x	x		x	x	x
*Streptomyces*	x (prodiginines)	x	x	x	x	x
*Pseudomonas*		x	x	x	x	x
*Serratia*	x	x				x
**FUNGI**						
*Pythium*	x	x				x
*Fusarium*	x (VOCs)^1^	x	x	x (DAMP release)^2^	x	x
*Trichoderma*	x	x			x	x
*Verticillium*		x	x	x (DAMP release)		
*Talaromyces*	x				x	x
*Penicillium*		x		x (DAMP release)	x	x
*Muscodor*	x (VOCs)					
*Gliocladium*	x	x			x	x
Mycorrhizae		x		x		

1 *VOCs: volatile compounds*.

2 *DAMP: damage associated molecular pattern*.

#### Reducing germination of inoculum

Especially in the case of a monocyclic disease such as Verticillium wilt, reducing the germination of primary inoculum is an interesting mode of action of potential BCAs. Root application of the BCAs *P. alvei* K-165, *Arthrobacter* sp. FP15 and *Blastobotrys* sp. FP12 resulted in the reduction of microsclerotia germination of *V. dahliae* in the rhizosphere of eggplants (Antonopoulos et al., 2008; Papasotiriou et al., 2013). Al-Rawahi and Hancock (1998) furthermore demonstrated that *P. oligandrum* was able to parasitize *V. dahliae* and to impede its microsclerotia formation. Interestingly, the BCA *T. flavus* decreased the viability of *V. dahliae* microsclerotia on senescent potato stems, which eventually could limit the release of these surviving structures to the soil (Nagtzaam et al., 1998). Mycofumigation with the volatile organic compounds of *Muscodor albus, Muscodor roseus*, and *F. oxysporum* CanR-46 also effectively reduced inoculum density of *V. dahliae* in the soil, thereby suppressing Verticillium wilt in respectively eggplant and cotton (Stinson et al., 2003; Zhang et al., 2015). In addition, the iturins of the culture filtrate of *B. amyloliquefaciens* 41B-1 suppressed *V. dahliae* microsclerotial germination, while the prodiginines produced by *S. lividans* reduced the formation of *V. dahliae* microsclerotia (Meschke et al., 2012; Han et al., 2015). The importance of biosurfactant production in the suppression of *Verticillium* microsclerotia viability by *Pseudomonas* spp. has only been shown *in vitro* (Debode et al., 2007). The germination of *V. dahliae* microsclerotia was also reduced by several *Gliocladium roseum* strains (Keinath et al., 1991; Varo et al., 2016b). Remarkably, effects of BCAs on surviving mycelium and chlamydospores were not reported. A possible explanation is that almost all BCAs have been tested against *V. dahliae* and *V. longisporum*, which only form microsclerotia to survive in soil (Table 1).

#### Growth promotion

BCAs of *Verticillium* often promote root and/or shoot growth and this has been reported for isolates of the bacterial genera *Bacillus, Paenibacillus, Streptomyces, Enterobacter, Pseudomonas*, and *Serratia*, and the fungal(-like) genera *Pythium, Fusarium, Nectria, Trichoderma, Verticillium, Penicillium, Phomopsis*, and AMF. The plant growth promoting effect of BCAs can counteract the adverse effect of pathogenic *Verticillium* species on the yield of crops as exemplified by the interaction of *S. plymuthica* R12 and *V. dahliae* in strawberry. Although treatment of strawberry with this *Serratia* strain resulted in a higher disease incidence of Verticillium wilt, a five-fold enhancement of the number of stolons and a yield enhancement of more than 70% was found (Berg et al., 2001). Production of plant growth hormones may be involved in improving plant growth mediated by the BCAs. Auxin production was demonstrated *in vitro* for some bacterial BCAs such as *B. amyloliquefaciens* 5-127, *P. alvei* K-165, and *S. plymuthica* HRO-C48 (Kalbe et al., 1996; Tjamos et al., 2004). Besides mechanisms involving phytohormones, enhanced growth may also be exerted by improved nutrient acquisition (Berg, 2009). Soil inoculation with a consortium of three plant-growth promoting rhizobacteria, active against *Verticillium* in cotton, improved soil properties in field experiments, including an increase in organic matter and the availability of nitrogen, phosphorus and potassium (Yang et al., 2014). AMF are known to promote plant growth and several of them reduce Verticillium wilt in solanaceous plants and alfalfa (Hwang et al., 1992; Liu, 1995; Matsubara et al., 1995; Karagiannidis et al., 2002; Garmendia et al., 2004a,b,c, 2006; Demir et al., 2015). Treatment with *Funneliformes mosseae* resulted in a higher phosphorus and nitrogen uptake in tomato and eggplant (Karagiannidis et al., 2002). Also pepper plants associated with *G. deserticola* had a higher phosphorus uptake (Garmendia et al., 2004b). This increased capacity for nutrient uptake could contribute to diminish the deleterious effect of the pathogen (Karagiannidis et al., 2002; Garmendia et al., 2004b).

#### Competition

Competition for space, infection sites and nutrients is well-established as working mechanism of BCAs and was suggested to be involved in the interaction between *Verticillium* and several biocontrol isolates of *Bacillus, Streptomyces, Pseudomonas, Verticillium*, and *Fusarium*. For *Verticillium*, particularly competition for nutrients and/or infection sites in the soil and in/on the roots may be an efficient mode of action in controlling the disease. It is expected that bacterial BCAs compete for nutrients and infection sites in the rhizosphere and cortex, while BCAs such as *Verticillium* and *Fusarium* can also colonize the xylem and occupy the same niche as *Verticillium*. A commonly cited example of competition is that for iron. Under iron-limiting conditions, bacteria produce siderophores with high affinity for ferric iron. By binding available iron these bacteria prevent the pathogens' access to the limited pool of soluble iron in the rhizosphere and in that way the growth of the pathogen is hindered (Loper and Buyer, 1991; Loper and Henkels, 1999). The *in vitro* production of siderophores was shown for a number of BCAs with antagonistic effect on *Verticillium* (Berg et al., 1996, 2000; Mercado-Blanco et al., 2004; Li et al., 2010; Xue et al., 2013). However, Maldonado-González et al. (2015a,b) showed that siderophore production is not required for biological control of Verticillium wilt by *Pseudomonas fluorescens* PICF7.

#### Induced resistance

Induced resistance has frequently been proposed to be part of the working mechanism of the BCAs. Evidence of triggering plant defense responses was provided for antagonistic isolates of the bacterial genera *Arthrobacter, Bacillus, Paenibacillus, Streptomyces*, and *Pseudomonas*, and of the fungal genera *Fusarium, Verticillium, Penicillium, Blastobotrys, Coriolopsis*, and *Trametes*. Also AMF of the genera *Glomus, Gigaspora* and *Claroideoglomus* were able to induce resistance. *P. alvei* K-165 and *F. oxysporum* F2 induced the expression of defense-related genes *PR1* and *PR4* in eggplant. Moreover, the expression of these genes was positively correlated with the rhizosphere population of both BCAs (Angelopoulou et al., 2014). In *Arabidopsis*, it has been shown that the resistance induced by *P. alvei* K-165 against *V. dahliae* is dependent on both salicylate and jasmonate-dependent defense pathways (Tjamos et al., 2005; Gkizi et al., 2016). Results of a split-root experiment indicated the involvement of induced resistance in the protection of eggplant against *V. dahliae* by *Arthrobacter* sp. FP15 and *Blastobotrys* sp. FP12 (Papasotiriou et al., 2013). The endophytic BCA *P. fluorescens* PICF7 has been shown to activate an array of defense pathways in the roots and aerial tissues of olive upon colonization of the roots (Schilirò et al., 2012; Gómez-Lama Cabanás et al., 2014). Recently, Gómez-Lama Cabanás et al. (2017) demonstrated that the expression of defense-related genes differed depending on whether or not *V. dahliae* and *P. fluorescens* PICF7 colonized the same sectors of the roots of olive plants. Interestingly, no biocontrol was observed when *V. dahliae* and *P. fluorescens* PICF7 were spatially separated. In the case of *B. amyloliquefaciens* 41B-1, iturins could induce plant defense responses and mediate pathogen-associated molecular pattern (PAMP)-triggered immunity against *V. dahliae* in cotton (Han et al., 2015). Applying exudates of several saprobe fungi (*Coriolopsis rigida, Trametes versicolor, F. lateritium, P. chrysogenum*, and the non-pathogenic *V. dahliae*-2379) could control *V. dahliae* disease of tomato probably through hydrolyzing root cell wall components. This generates damage associated patterns (DAMPs) which could act as elicitors of plant defense (García et al., 2011). PAMPs and DAMPs can be recognized by specific membrane-bound receptors in the plant, leading to PAMP-triggered immunity (PTI; Boller and Felix, 2009; Zipfel, 2014). Induced resistance by AMF resulted in a more balanced antioxidant metabolism (Garmendia et al., 2004a), the induction of defense-related enzymes (Garmendia et al., 2006) and accumulation of lignin in the roots (Matsubara et al., 1995).

#### What about cross-protection?

The protection of plants against virulent *Verticillium* spp. by closely related isolates that are non-pathogenic on that specific host has often been described as cross-protection. Only in a few studies the underlying mechanisms of this phenomenon were elucidated (Shittu et al., 2009; García et al., 2011). Mechanisms involved include induced resistance, competition for space (including infection sites) and nutrients, and plant growth promotion. *In vitro*, it was often shown that neither isolate is inhibitory to the other. The best protection is accomplished if the protective isolates are applied to the plants before challenge treatment with the pathogen. Also the concentrations of inoculum of both the pathogen and the beneficial organism are of importance for the level of control (Shittu et al., 2009; Tyvaert et al., 2014). *Verticillium* species have proven to expand their host range and the stability of the interaction between non-pathogenic and pathogenic isolates remains an open question (Shittu et al., 2009).

#### What about antibiosis and mycoparasitism of verticillium mycelium?

The majority of BCAs included in this study showed *in vitro* antagonism against *Verticillium* mycelium (Tables 2, 3, 4) but a possible role of antibiosis in biocontrol *in planta* has not been demonstrated. Only when production at the site of biocontrol is demonstrated or when activity is proved by the use of non-producing or over-producing mutants, or reporter strains, the role of metabolites in disease biocontrol can be confirmed (Whipps and McQuilken, 2009). To our knowledge, these types of studies have not been reported for *Verticillium* biocontrol. Another type of direct antagonism is mycoparasitism and the associated production of extracellular lytic enzymes. Chitinases, proteases, and glucanases are produced *in vitro* by many of the studied BCAs of *Verticillium*, but clear evidence that these enzymes play a role in the direct interaction with the pathogen in the presence of plants is lacking. Regarding the life cycle of *Verticillium*, germination of survival structures such as microsclerotia is stimulated by the direct vicinity of germinating seeds or plant roots. Root penetration and subsequent colonization of the xylem vessels can be achieved within only 2–4 days (Heinz et al., 1998; Chen et al., 2004; Fradin and Thomma, 2006). Possibilities for reducing mycelial growth in the rhizosphere by direct antagonism may therefore be limited. Direct antagonism *in planta* is only possible for those BCAs that are able to colonize the cortex or xylem. The production of antibiotics and inhibitory metabolites is influenced by plant type and age, nutrient availability, environmental conditions, microorganisms present and the pathogen itself (Molina et al., 2003; Duffy et al., 2004; Maurhofer et al., 2004; Morello et al., 2004; Compant et al., 2005). It is not clear if conditions inside the plant are conducive for the production of antimicrobial compounds. *In planta* studies on the behavior of BCAs are limited but for *T. harzianum*, the interaction with *V. dahliae* in olive was investigated. Mycoparasitism of *V. dahliae* by *T. harzianum* occurred *in vitro*, although there was no evidence that this also happens *in planta* (Ruano-Rosa et al., 2016). In this context, it is interesting to notice that control of *Verticillium* by *Trichoderma*, for which the main modes of action include antibiosis and mycoparasitism, is limited. *Trichoderma* is one of the most studied and successful BCAs, with many commercial products that are used in practice to control a variety of soil-borne pathogens such as *Rhizoctonia, Fusarium, Sclerotinia, Botrytis*, and *Pythium*. Possibly, *Trichoderma* strains were originally selected for control of other soil-borne pathogens and were later on tested against *Verticillium*. Therefore, not the best strains for biocontrol of *Verticillium* might have been selected. Interestingly, it was shown by Carrero-Carrón et al. (2016) that *T. asperellum* T25 that was effective in controlling Verticillium disease in olive had the highest ability to grow endophytically in the roots. But in comparison with other isolates, it had the lowest inhibitory effect on the *in vitro* growth of *V. dahliae*. The capacity of a biocontrol strain to compete for the same ecological niche of *Verticillium* could be crucial, indicating that selection criteria should not focus on *in vitro* antagonism.

## What are the key factors in the process from selection of the BCA to successful implementation?

From our survey of biocontrol studies we can conclude that common BCAs such as *Trichoderma, Pythium, Gliocladium*, and AMF are not the best candidates for augmentative biological control of Verticillium wilt. Few studies reported the biocontrol effect of *Gliocladium* on Verticillium wilt. Some *Gliocladium* strains could reduce microsclerotia viability in soil conditions, but the number of reports about successful biocontrol *in planta* is limited (Keinath et al., 1991; Varo et al., 2016b). The biopesticide Polyversum®, containing *P. oligandrum*, showed no control of *Verticillium* in one study and in another study, it resulted in variable control (Al-Rawahi and Hancock, 1998; Rekanovic et al., 2007). Some of the *Trichoderma* strains (*T. asperellum* T34, *T. harzianum* T-22) were shown to be able to reduce Fusarium wilt (Cotxarrera et al., 2002; Gilardi et al., 2007; Sant et al., 2010) and are approved by the EU as biopesticide against *Fusarium* but not against *Verticillium*. It would be expected that *F. oxysporum* and *Verticillium* can be controlled by the same BCAs because they have apparently similar characteristics. Both pathogens share the same ecological niche: they are soil-borne pathogens able to colonize the vascular system with the production of similar symptoms. A closer look to the infection and colonization process gives evidence for some important differences. *Verticillium* inhabits the lower parts of the plant for a longer time than *F. oxysporum* (Klimes et al., 2015). *F. oxysporum* has a higher degree of host specialization and produces symptoms faster (Klosterman et al., 2011). The *V. dahliae* enzyme VdThi4, required for biosynthesis of a thiamine (vitamin B1), has been shown to play a role in the colonization process. *VdThi4* deletion mutants are unable to colonize the upper portion of the plant. In *F. oxysporum*, however, the *VdThi4* homolog s*tri35* was not required for virulence (Hoppenau et al., 2014). Tomato plant cells respond differently to infection by both pathogens (Ferraris et al., 1974; Cooper and Wood, 1980; Bishop and Cooper, 1983a,b). Recently, genomic insights into both pathogens revealed some differences in the secretome. More specifically, a protein family involved in attachment to plant cell walls and increase of enzyme efficiency was expanded in *Verticillium* (Klosterman et al., 2011). These differences may explain why some BCAs are effective against *Fusarium* but not against *Verticillium*.

### Where to look for potential BCAs?

Disease suppressive soils are an interesting source of BCAs with potential against soil-borne diseases (Cook, 1985). *Fusarium* suppressive soils have extensively been studied while soil suppressiveness for *Verticillium* is rarely reported. A strain of *F. oxysporum* (Fo47) originated from suppressive soils for Fusarium wilt of tomato and had also biocontrol activity against Verticillium wilt on pepper (Veloso and Díaz, 2012). Keinath and Fravel (1992) demonstrated that by successive croppings, some soils exhibit induced suppressiveness to Verticillium wilt of potato. Only a few studies were carried out with isolates from suppressive soils for Verticillium wilt of potato and cauliflower. From these soils non-pathogenic *Verticillium* isolates, belonging to *V. tricorpus* and *V. isaacii*, were obtained that could control Verticillium wilt in potato and cauliflower (Davis et al., 2000; França et al., 2013; Tyvaert et al., 2014).

Organic amendments have proven to be disease suppressive and are therefore interesting reservoirs of potential BCAs. Several isolates controlling Verticillium wilt were obtained from suppressive composts: two *F. oxysporum* and two *P. fluorescens* isolates originated from the rhizosphere of eggplants grown in soil amended with disease suppressive compost (Malandraki et al., 2008), while the isolates belonging to *Arthrobacter* and *Blastobotrys* were obtained from disease suppressive olive mill compost (Papasotiriou et al., 2013). Another strategy to look for successful BCAs is to identify healthy plants in infested fields. In this way a *Nectria* isolate and two *B. subtilis* isolates with biocontrol activity against *Verticillium* were recovered from healthy cotton roots in infested soil (Luo et al., 2010; Zheng et al., 2011; Li et al., 2013). Most of the other bacterial BCAs described in Table 2 were obtained from the rhizosphere or roots of host plants. The origin of the fungal BCAs described in Table 3 is not always indicated. Clearly, not a lot of the studied isolates were obtained from sources giving already some evidence for biological control. It does not necessarily mean that those isolates perform better but at least they are expected to establish better in field conditions, as they are able to colonize the soil or host plants.

### Desirable characteristics

The ability to affect surviving structures of *Verticillium* by antibiosis or mycoparasitism is a desirable trait of BCAs resulting in a reduction of the primary inoculum. Selection of BCAs sharing the same ecological niche as *Verticillium* is promising, since these organisms can compete with *Verticillium* for infection sites, space and nutrients. For instance in the tripartite interaction *V. dahliae*-olive-*P. fluorescens* PICF7, niche overlap between the BCA and the pathogen *in planta* was necessary for effective biocontrol (Gómez-Lama Cabanás et al., 2017). Efficient root colonizers can compete with *Verticillium* for infection sites. In addition, they may protect the plant by triggering induced resistance by secreting PAMPs or releasing DAMPs from plant cells. BCAs with an endophytic lifestyle that colonize the cortex and/or the xylem are protected against adverse environmental conditions, and can exclude *Verticillium* from the same niche by competition for space and nutrients, as exemplified by a non-pathogenic *F. oxysporum* (Pantelides et al., 2009), or by inducing resistance responses in the plant as shown for *Bacillus* spp. (Han et al., 2015). Often, non-pathogenic fungi that are closely related to the pathogen can successfully control disease in naturally infested soils (Herr, 1995; Gutteridge et al., 2007; Alabouvette et al., 2009). In the case of Verticillium wilt this has been demonstrated for non-pathogenic *Verticillium* isolates. However, it is important to confirm that these isolates are really non-pathogenic on a wide range of plants. Finally, the ability to promote plant growth can compensate for some of the deleterious effects caused by pathogenic *Verticillium* spp. *In vitro* screening for antimicrobial activity against *Verticillium* mycelium correlates poorly or not at all with biocontrol activity *in planta* and does not seem to be the best strategy to look for good *Verticillium* BCAs.

The ability to control *Verticillium* in several host plants or to control other soil-borne and/or vascular pathogens, is interesting to increase the market potential of the BCA. Several BCAs able to reduce Verticillium disease were also effective in controlling other diseases and examples are summarized hereafter. Non-pathogenic *F. oxysporum* isolates also controlled Fusarium wilt and *Phytophthora* root rot and blight of pepper plants (Díaz et al., 2005; Veloso and Díaz, 2012). Cotton plants treated with dry mycelium of *P. chrysogenum* exhibited reduced symptoms of *Verticillium* and Fusarium wilt (Dong et al., 2006; Zhang et al., 2011). Mycofumigation with *Muscodor* spp. could control seedling diseases of sugar beet next to Verticillium wilt of eggplant (Stinson et al., 2003). Besides its biocontrol effect on *V. dahliae* in eggplant and potato, the bacterial BCA *P. alvei* K-165 reduced root discoloration and hypocotyl lesions caused by the black root rot fungus *Thielaviopsis basicola* on cotton seedlings (Tjamos et al., 2004; Schoina et al., 2011). *Pseudomonas chlororaphis* MA 342, which suppressed *V. longisporum* in oilseed rape, furthermore controls a wide range of cereal seed-borne diseases and is the active organism in the registered products Cedomon® and Cerall® (Johnsson et al., 1998; Abuamsha et al., 2011).

Omics technologies are an interesting tool for the selection of promising BCAs, as these technologies allow in-depth characterization of the strain. The modes of action of a BCA can be identified by characterization of genes, mRNAs, and proteins. Also the properties of strains with different control efficacy can be compared. This may lead to the selection of BCAs with the best control potential in terms of efficacy and consistency (Massart et al., 2015).

### Evaluation of biocontrol activity

Experiments with BCAs are often carried out in sterile soils using plants that have been artificially inoculated with *Verticillium* via root dipping in a conidial suspension or via soil drench with a conidial suspension. These experimental conditions are quite different from natural infested field conditions. First of all, in sterile soils, the BCA can easily establish, while BCAs often fail to work in the field due to more complex conditions. Secondly, disease development in sterile soils is fast and often leads to severe symptoms. This can be a disadvantage for the BCA and possibly some effective BCAs are not selected because they seem of minor importance during the selection procedure in sterile conditions. Preferentially, experiments should be carried out with naturally infested soil, in field and greenhouse conditions, or by using microsclerotia as primary inoculum. In addition, the plants should be observed until the onset of flowering as the spread of *Verticillium* in the host tissue has been suggested to be induced by the initiation of flowering (Veronese et al., 2003; Zhou et al., 2006). Also screening for BCAs that target the primary inoculum should be done in conditions that mimic the natural situation. For instance, *Microsphaeropsis ochracea* reduced the microsclerotia viability in sterile soils but not in unsterile soils and failed to control Verticillium wilt of oilseed rape in the field (Stadler and von Tiedemann, 2014). It is therefore interesting to start screening for biocontrol strains from the field, to perform subsequently experiments in controlled conditions and to go back to the field finally.

### Formulation and application

In order to develop a promising BCA into a commercial product, large scale production, formulation, preservation conditions, shelf life, and application methods should be investigated. Nowadays, researchers interested in biocontrol are becoming more aware of the importance of these issues in product development.

Fungi and bacteria that produce surviving structures are interesting because these structures can be used as the active substance of the biocontrol product. Usually they are persistent to adverse environmental conditions and can be preserved and distributed without special requirements. Therefore, sporulating Gram-positive microorganisms, such as *Bacillus* and *Streptomyces*, are preferred rather than Gram-negative bacteria. Soil-borne fungi usually produce surviving structures such as chlamydospores in the case of *F. oxysporum* and microsclerotia in case of *Verticillium* species. A possible disadvantage of surviving structures is that the production process might be complex leading to a higher cost. Also the ability of those BCAs to become persistent in the new environment should be considered. The capacity of a strain to produce different structures is a desirable characteristic for application in different crop systems.

Application of the *Verticillium* BCAs close to the roots, where *Verticillium* initially infect the plants, could be the most effective strategy. The early introduction of the BCA by seed treatment and treatment of seedlings at the nursery stage could provide better relief from subsequent *Verticillium* infection than when the BCA is applied directly to the field. In the case of seed treatment, compatibility with standard seed treatments should be ensured. BCAs that can reduce germination of primary inoculum could be added to compost amendments or to the substrate.

Combining two or more BCAs is another interesting approach to improve the efficacy of biocontrol or to control different pathogens and even pests. Therefore, the application of the specific isolates should be compatible without reducing their single effect. Yang et al. (2013) showed that the combined application of three endophytic bacterial strains resulted in a better biocontrol efficacy of Verticillium wilt in cotton than their individual applications, which was probably linked to the fact that the different strains are predominant in different developmental stages of cotton. Also the application of a consortium of three rhizobacteria, *Bacillus cereus* AR156, *B. subtilis* SM21 and *Serratia* sp. XY21, resulted in higher biocontrol efficacy against Verticillium wilt in cotton compared to the individual strains (Yang et al., 2014). For other plant pathogens, it has been shown that mixtures of bacterial and fungal BCAs are more effective in controlling diseases such as *Rhizoctonia* and *Pythium* (Colla et al., 2012). The strength of a mixture is that BCAs can be combined that interact in a different way with the pathogen and/or the plant. Moreover, if conditions are not favorable for one of the BCAs, the other can take over. The drawback is that all isolates used in the mixture need to be registered.

The reliability of a product based on microbial BCAs is a crucial issue in ensuring long-term acceptance and sustained use by farmers. Standardized guidelines for quality control of the (potential) commercially available BCAs may help to avoid failures in their practical application and to prevent the application of organisms with detrimental effects. Parameters to be considered include content of fertilizers, presence of contaminants, traceability of the origin of the BCA, possible allelopathic effects of the BCA on the germination of some plant species and effectiveness under various conditions.

As Verticillium wilt is an emerging problem in different crops, some agricultural systems seem to promote Verticillium disease. Therefore, it could be difficult to reach satisfactory levels of control of Verticillium with a BCA in such a system. To implement biocontrol as a tool of IPM in agriculture, the current approach should be changed to a holistic management (van Lenteren et al., 2017).

## Conclusion

The application of BCAs is an interesting building block of sustainable and environmentally sound management strategies of Verticillium wilt. A holistic management should be considered to reach satisfactory levels of control by a BCA. Based on the number of currently known isolates with biocontrol activity against *Verticillium* species, the predominant genera are *Pseudomonas, Bacillus, Fusarium*, and *Verticillium*. Particularly soils or organic amendments suppressive for *Verticillium* disease and healthy plants in infested fields are attractive spots to find (new) BCAs of *Verticillium*. The ability to affect survival structures, sharing the same ecological niche as *Verticillium*, inducing resistance responses in the plant and promoting plant growth are desirable characteristics of a competent BCA against Verticillium wilt. Evaluating the biocontrol efficacy of BCAs in conditions that mimic the field situation is expected to significantly improve the chance of successful application in practice. In order to facilitate the further commercialization of a promising BCA of *Verticillium*, potential bottlenecks such as large-scale production, formulation, preservation conditions, shelf life, and application methods, should be tackled early in the selection process.

## Author contributions

SD wrote the part about bacteria involved in biocontrol against Verticillium and made the figures. LT wrote the part about fungi involved in biocontrol against Verticillium and helped in making the figures. SD and LT contributed equally. SF and MH revised the manuscript and helped in structuring and editing the work.

### Conflict of interest statement

The authors declare that the research was conducted in the absence of any commercial or financial relationships that could be construed as a potential conflict of interest.
